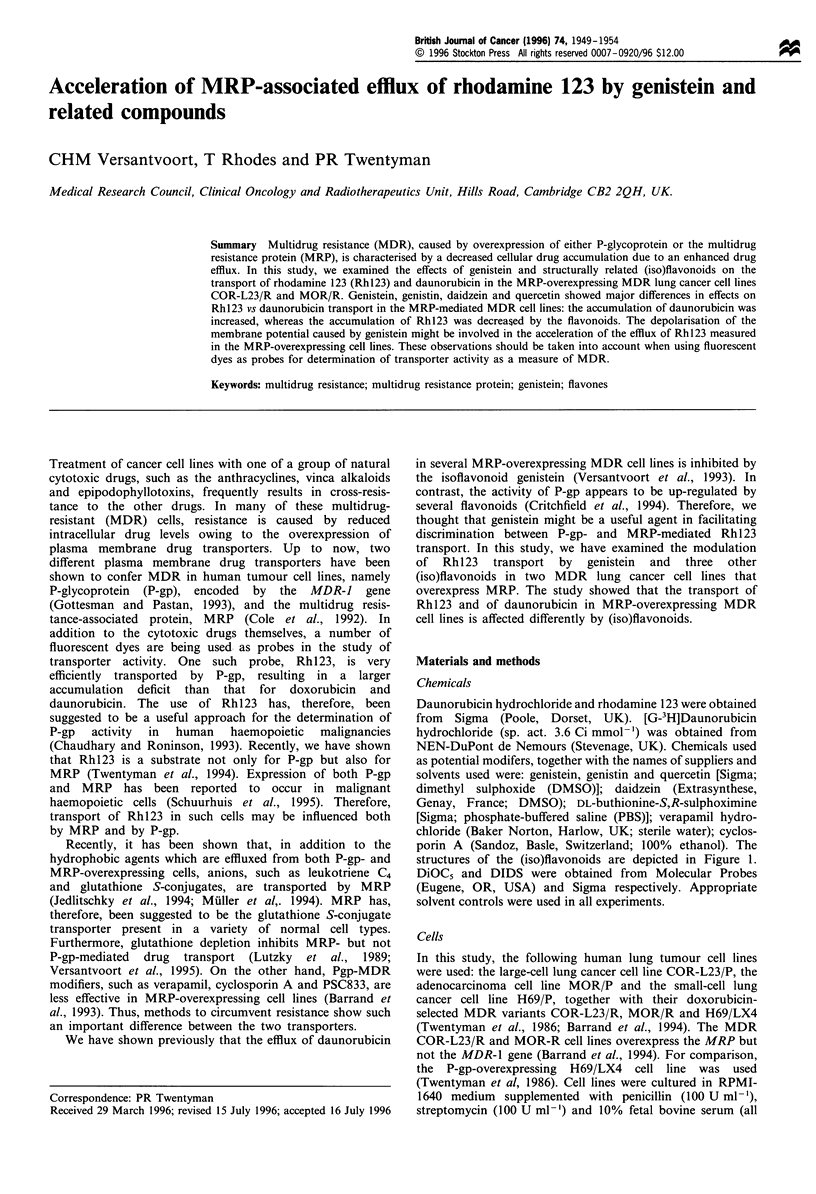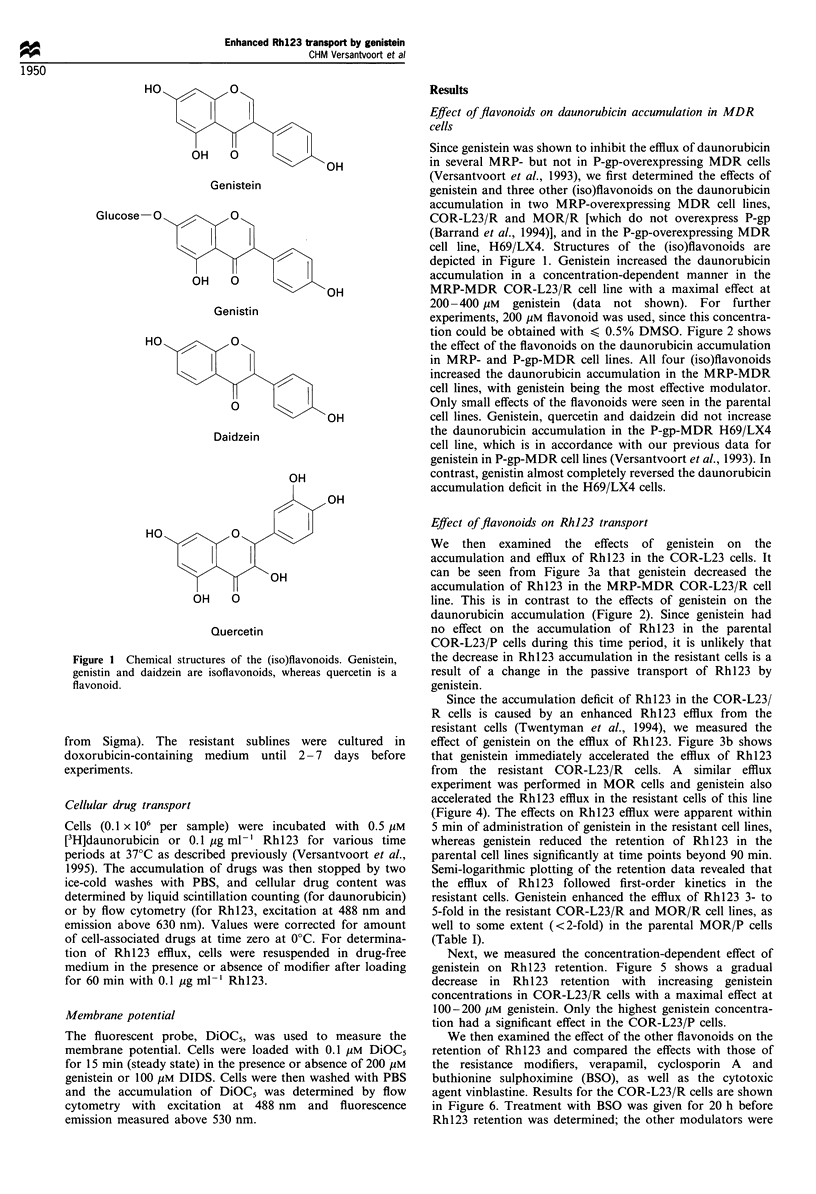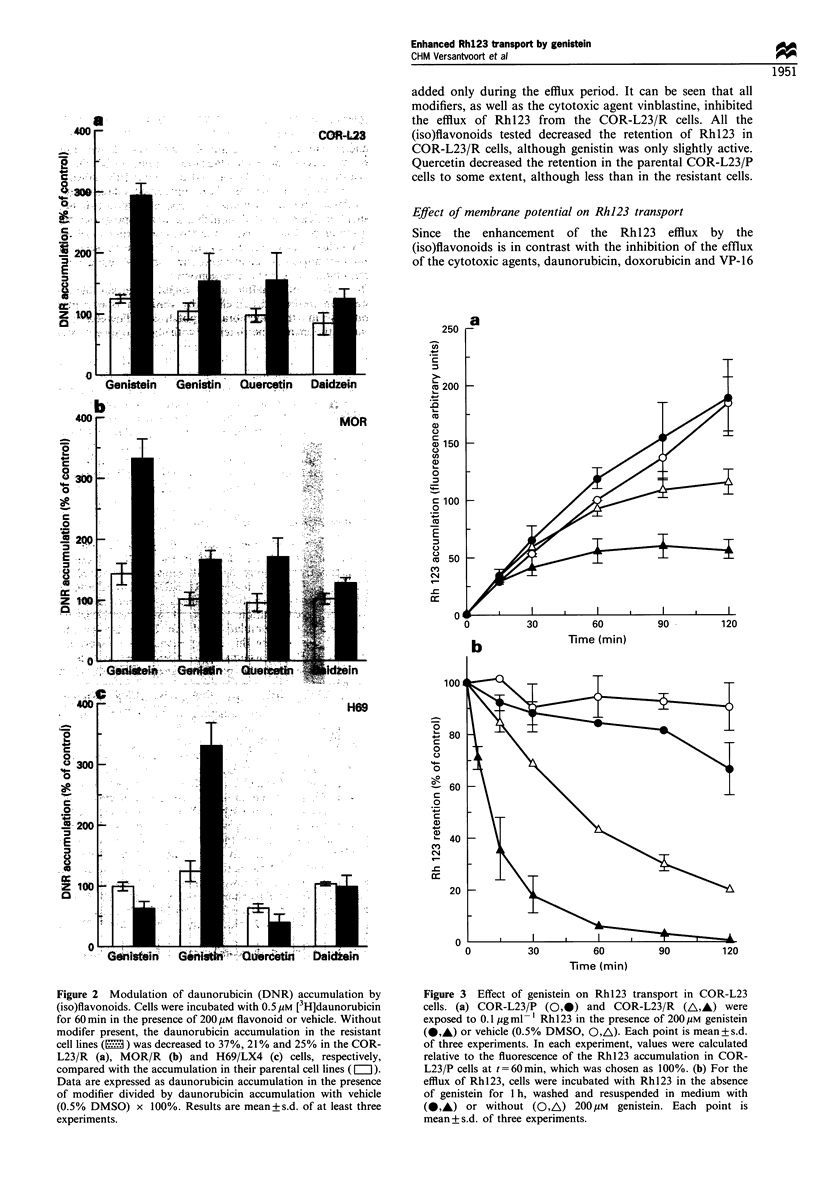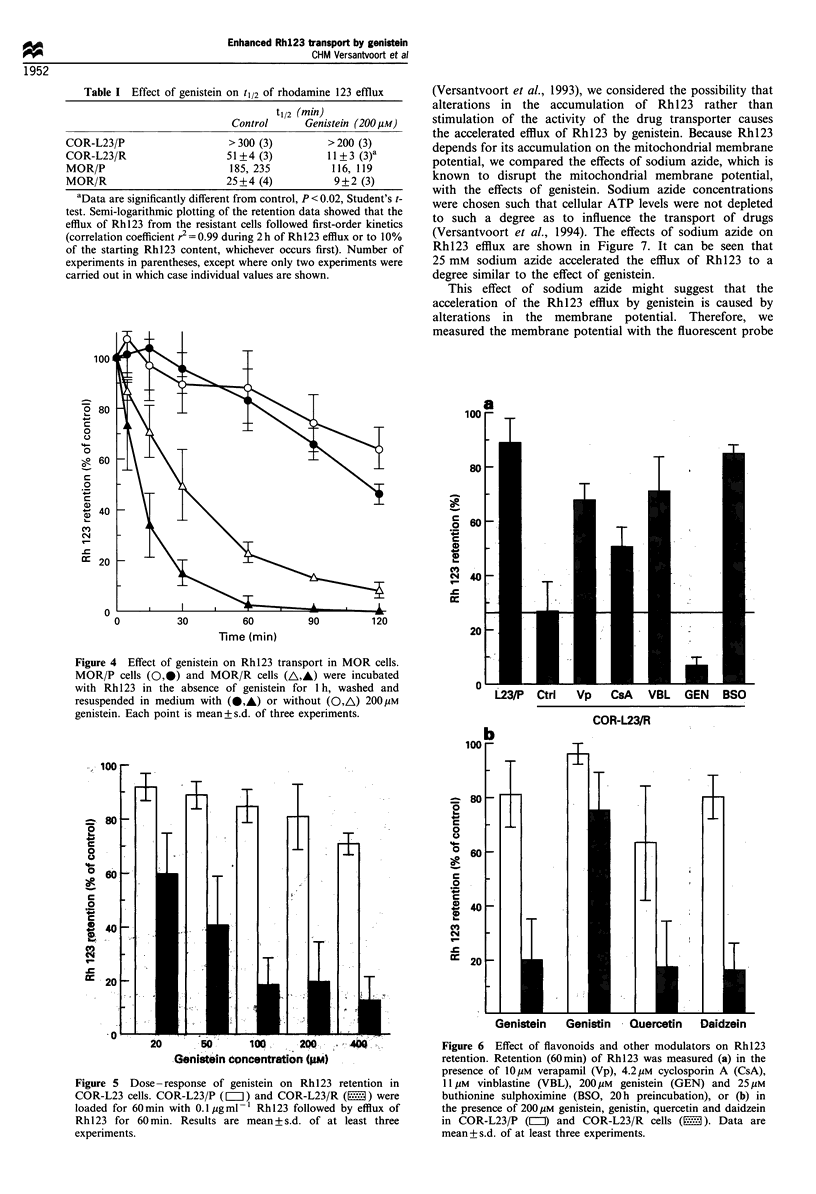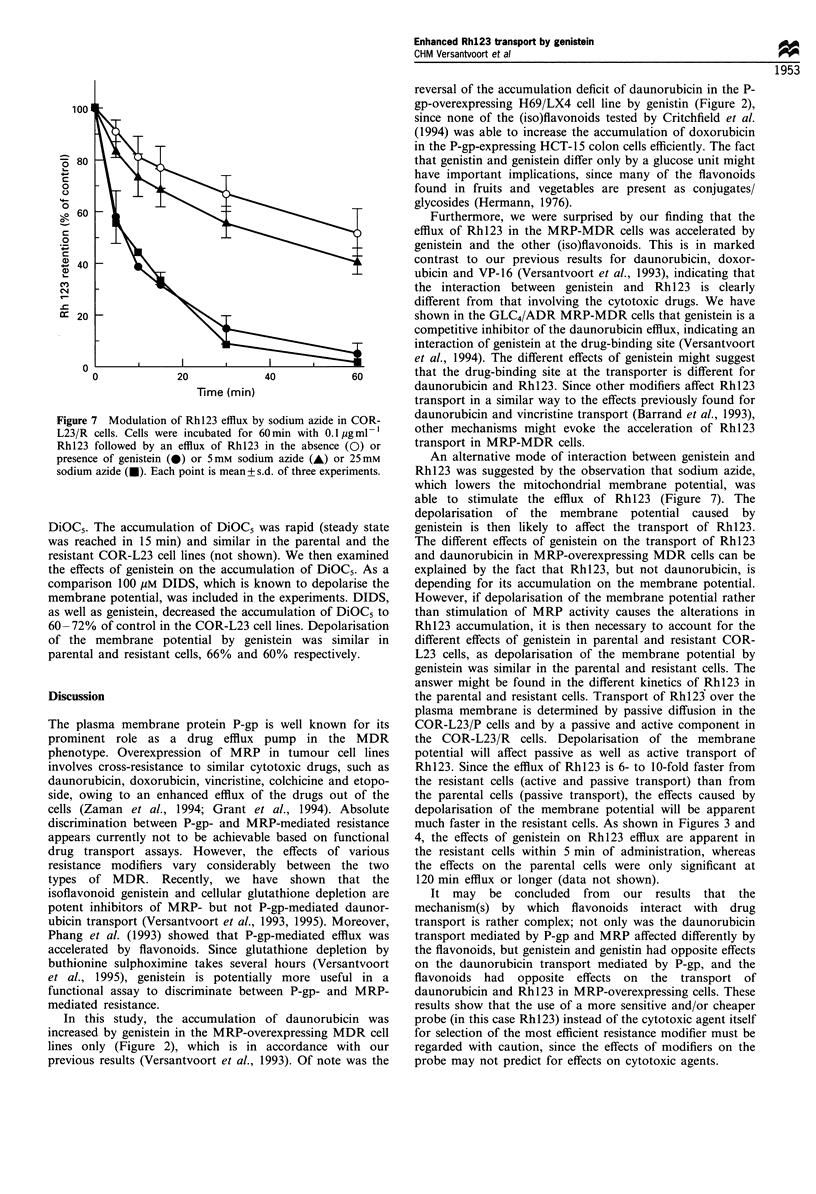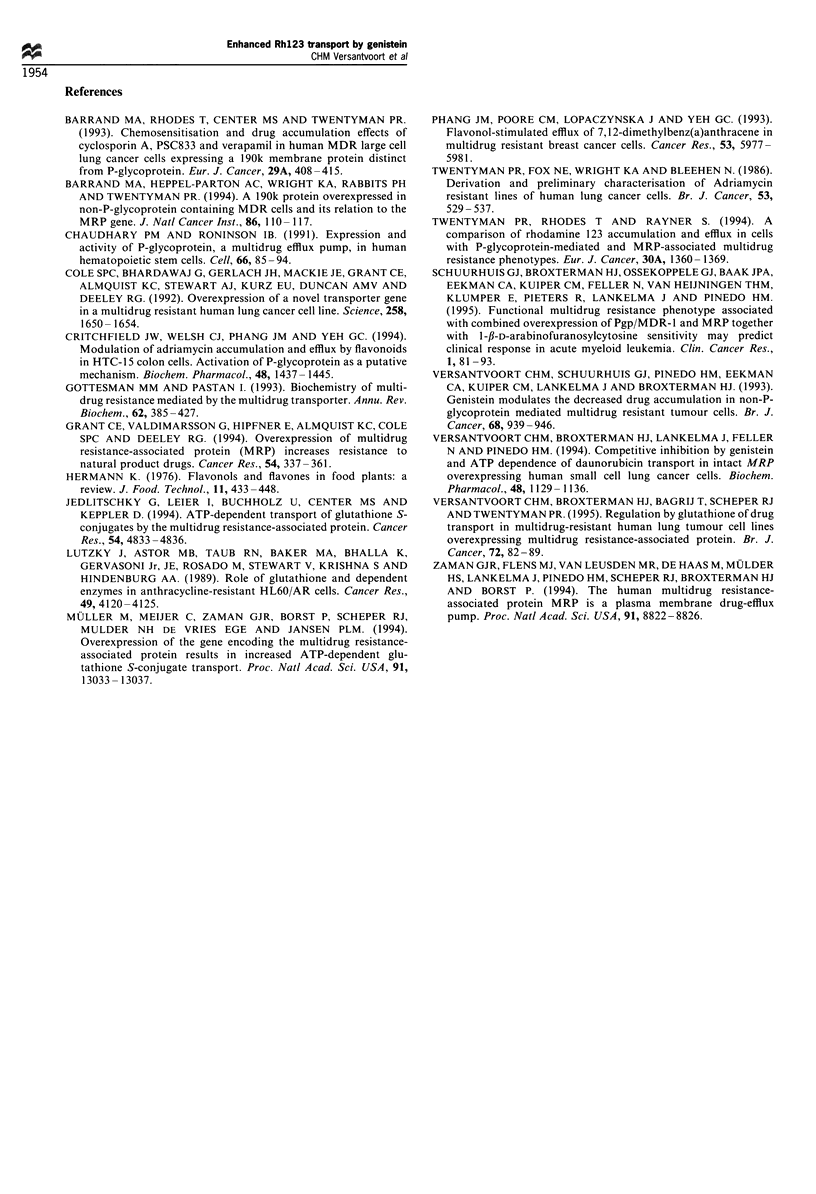# Acceleration of MRP-associated efflux of rhodamine 123 by genistein and related compounds.

**DOI:** 10.1038/bjc.1996.658

**Published:** 1996-12

**Authors:** C. H. Versantvoort, T. Rhodes, P. R. Twentyman

**Affiliations:** Medical Research Council, Clinical Oncology and Radiotherapeutics Unit, Cambridge, UK.

## Abstract

Multidrug resistance (MDR), caused by overexpression of either P-glycoprotein or the multidrug resistance protein (MRP), is characterised by a decreased cellular drug accumulation due to an enhanced drug efflux. In this study, we examined the effects of genistein and structurally related (iso)flavonoids on the transport of rhodamine 123 (Rh123) and daunorubicin in the MRP-overexpressing MDR lung cancer cell lines COR-L23/R and MOR/R. Genistein, genistin, daidzein and quercetin showed major differences in effects on Rh123 vs daunorubicin transport in the MRP-mediated MDR cell lines: the accumulation of daunorubicin was increased, whereas the accumulation of Rh123 was decreased by the flavonoids. The depolarisation of the membrane potential caused by genistein might be involved in the acceleration of the efflux of Rh123 measured in the MRP-overexpressing cell lines. These observations should be taken into account when using fluorescent dyes as probes for determination of transporter activity as a measure of MDR.


British Journal of Cancer (1996) 74, 1949–1954
© 1996 Stockton Press All rights reserved 0007–0920/96 $12.00

### Acceleration of MRP-associated efflux of rhodamine 123 by genistein and related compounds

CHM Versantvoort, T Rhodes and PR Twentyman

*Medical Research Council, Clinical Oncology and Radiotherapeutics Unit, Hills Road, Cambridge CB2 2QH, UK.*

**Summary** Multidrug resistance (MDR), caused by overexpression of either P-glycoprotein or the multidrug resistance protein (MRP), is characterised by a decreased cellular drug accumulation due to an enhanced drug efflux. In this study, we examined the effects of genistein and structurally related (iso)flavonoids on the transport of rhodamine 123 (Rh123) and daunorubicin in the MRP-overexpressing MDR lung cancer cell lines COR-L23/R and MOR/R. Genistein, genistin, daidzein and quercetin showed major differences in effects on Rh123 *vs* daunorubicin transport in the MRP-mediated MDR cell lines: the accumulation of daunorubicin was increased, whereas the accumulation of Rh123 was decreased by the flavonoids. The depolarisation of the membrane potential caused by genistein might be involved in the acceleration of the efflux of Rh123 measured in the MRP-overexpressing cell lines. These observations should be taken into account when using fluorescent dyes as probes for determination of transporter activity as a measure of MDR.

**Keywords:** multidrug resistance; multidrug resistance protein; genistein; flavones

Treatment of cancer cell lines with one of a group of natural cytotoxic drugs, such as the anthracyclines, vinca alkaloids and epipodophyllotoxins, frequently results in cross-resistance to the other drugs. In many of these multidrug-resistant (MDR) cells, resistance is caused by reduced intracellular drug levels owing to the overexpression of plasma membrane drug transporters. Up to now, two different plasma membrane drug transporters have been shown to confer MDR in human tumour cell lines, namely P-glycoprotein (P-gp), encoded by the *MDR-1* gene (Gottesman and Pastan, 1993), and the multidrug resistance-associated protein, MRP (Cole *et al.*, 1992). In addition to the cytotoxic drugs themselves, a number of fluorescent dyes are being used as probes in the study of transporter activity. One such probe, Rh123, is very efficiently transported by P-gp, resulting in a larger accumulation deficit than that for doxorubicin and daunorubicin. The use of Rh123 has, therefore, been suggested to be a useful approach for the determination of P-gp activity in human haemopoietic malignancies (Chaudhary and Roninson, 1993). Recently, we have shown that Rh123 is a substrate not only for P-gp but also for MRP (Twentyman *et al.*, 1994). Expression of both P-gp and MRP has been reported to occur in malignant haemopoietic cells (Schuurhuis *et al.*, 1995). Therefore, transport of Rh123 in such cells may be influenced both by MRP and by P-gp.

Recently, it has been shown that, in addition to the hydrophobic agents which are effluxed from both P-gp- and MRP-overexpressing cells, anions, such as leukotriene $C_4$ and glutathione *S*-conjugates, are transported by MRP (Jedlitschky *et al.*, 1994; Müller *et al*,. 1994). MRP has, therefore, been suggested to be the glutathione *S*-conjugate transporter present in a variety of normal cell types. Furthermore, glutathione depletion inhibits MRP- but not P-gp-mediated drug transport (Lutzky *et al.*, 1989; Versantvoort *et al.*, 1995). On the other hand, Pgp-MDR modifiers, such as verapamil, cyclosporin A and PSC833, are less effective in MRP-overexpressing cell lines (Barrand *et al.*, 1993). Thus, methods to circumvent resistance show such an important difference between the two transporters.

We have shown previously that the efflux of daunorubicin in several MRP-overexpressing MDR cell lines is inhibited by the isoflavonoid genistein (Versantvoort *et al.*, 1993). In contrast, the activity of P-gp appears to be up-regulated by several flavonoids (Critchfield *et al.*, 1994). Therefore, we thought that genistein might be a useful agent in facilitating discrimination between P-gp- and MRP-mediated Rh123 transport. In this study, we have examined the modulation of Rh123 transport by genistein and three other (iso)flavonoids in two MDR lung cancer cell lines that overexpress MRP. The study showed that the transport of Rh123 and of daunorubicin in MRP-overexpressing MDR cell lines is affected differently by (iso)flavonoids.

### Materials and methods

### *Chemicals*

Daunorubicin hydrochloride and rhodamine 123 were obtained from Sigma (Poole, Dorset, UK). [G-$^3$H]Daunorubicin hydrochloride (sp. act. 3.6 Ci mmol$^{-1}$) was obtained from NEN-DuPont de Nemours (Stevenage, UK). Chemicals used as potential modifers, together with the names of suppliers and solvents used were: genistein, genistin and quercetin [Sigma; dimethyl sulphoxide (DMSO)]; daidzein (Extrasynthese, Genay, France; DMSO); DL-buthionine-*S*,*R*-sulphoximine [Sigma; phosphate-buffered saline (PBS)]; verapamil hydrochloride (Baker Norton, Harlow, UK; sterile water); cyclosporin A (Sandoz, Basle, Switzerland; 100% ethanol). The structures of the (iso)flavonoids are depicted in Figure 1. $DiOC_5$ and DIDS were obtained from Molecular Probes (Eugene, OR, USA) and Sigma respectively. Appropriate solvent controls were used in all experiments.

### *Cells*

In this study, the following human lung tumour cell lines were used: the large-cell lung cancer cell line COR-L23/P, the adenocarcinoma cell line MOR/P and the small-cell lung cancer cell line H69/P, together with their doxorubicin-selected MDR variants COR-L23/R, MOR/R and H69/LX4 (Twentyman *et al.*, 1986; Barrand *et al.*, 1994). The MDR COR-L23/R and MOR-R cell lines overexpress the *MRP* but not the *MDR*-1 gene (Barrand *et al.*, 1994). For comparison, the P-gp-overexpressing H69/LX4 cell line was used (Twentyman *et al*, 1986). Cell lines were cultured in RPMI-1640 medium supplemented with penicillin (100 U ml$^{-1}$), streptomycin (100 U ml$^{-1}$) and 10% fetal bovine serum (all

Correspondence: PR Twentyman
Received 29 March 1996; revised 15 July 1996; accepted 16 July 1996

Genistein

Genistin

Daidzein

Quercetin

**Figure 1** Chemical structures of the (iso)flavonoids. Genistein, genistin and daidzein are isoflavonoids, whereas quercetin is a flavonoid.

from Sigma). The resistant sublines were cultured in doxorubicin-containing medium until 2–7 days before experiments.

*Cellular drug transport*

Cells ($0.1 \times 10^6$ per sample) were incubated with 0.5 $\mu$M [$^3$H]daunorubicin or 0.1 $\mu$g ml$^{-1}$ Rh123 for various time periods at 37°C as described previously (Versantvoort *et al.*, 1995). The accumulation of drugs was then stopped by two ice-cold washes with PBS, and cellular drug content was determined by liquid scintillation counting (for daunorubicin) or by flow cytometry (for Rh123, excitation at 488 nm and emission above 630 nm). Values were corrected for amount of cell-associated drugs at time zero at 0°C. For determination of Rh123 efflux, cells were resuspended in drug-free medium in the presence or absence of modifier after loading for 60 min with 0.1 $\mu$g ml$^{-1}$ Rh123.

*Membrane potential*

The fluorescent probe, $DiOC_5$, was used to measure the membrane potential. Cells were loaded with 0.1 $\mu$M $DiOC_5$ for 15 min (steady state) in the presence or absence of 200 $\mu$M genistein or 100 $\mu$M DIDS. Cells were then washed with PBS and the accumulation of $DiOC_5$ was determined by flow cytometry with excitation at 488 nm and fluorescence emission measured above 530 nm.

### Results

*Effect of flavonoids on daunorubicin accumulation in MDR cells*

Since genistein was shown to inhibit the efflux of daunorubicin in several MRP- but not in P-gp-overexpressing MDR cells (Versantvoort *et al.*, 1993), we first determined the effects of genistein and three other (iso)flavonoids on the daunorubicin accumulation in two MRP-overexpressing MDR cell lines, COR-L23/R and MOR/R [which do not overexpress P-gp (Barrand *et al.*, 1994)], and in the P-gp-overexpressing MDR cell line, H69/LX4. Structures of the (iso)flavonoids are depicted in Figure 1. Genistein increased the daunorubicin accumulation in a concentration-dependent manner in the MRP-MDR COR-L23/R cell line with a maximal effect at 200–400 $\mu$M genistein (data not shown). For further experiments, 200 $\mu$M flavonoid was used, since this concentration could be obtained with $\leqslant$ 0.5% DMSO. Figure 2 shows the effect of the flavonoids on the daunorubicin accumulation in MRP- and P-gp-MDR cell lines. All four (iso)flavonoids increased the daunorubicin accumulation in the MRP-MDR cell lines, with genistein being the most effective modulator. Only small effects of the flavonoids were seen in the parental cell lines. Genistein, quercetin and daidzein did not increase the daunorubicin accumulation in the P-gp-MDR H69/LX4 cell line, which is in accordance with our previous data for genistein in P-gp-MDR cell lines (Versantvoort *et al.*, 1993). In contrast, genistin almost completely reversed the daunorubicin accumulation deficit in the H69/LX4 cells.

*Effect of flavonoids on Rh123 transport*

We then examined the effects of genistein on the accumulation and efflux of Rh123 in the COR-L23 cells. It can be seen from Figure 3a that genistein decreased the accumulation of Rh123 in the MRP-MDR COR-L23/R cell line. This is in contrast to the effects of genistein on the daunorubicin accumulation (Figure 2). Since genistein had no effect on the accumulation of Rh123 in the parental COR-L23/P cells during this time period, it is unlikely that the decrease in Rh123 accumulation in the resistant cells is a result of a change in the passive transport of Rh123 by genistein.

Since the accumulation deficit of Rh123 in the COR-L23/R cells is caused by an enhanced Rh123 efflux from the resistant cells (Twentyman *et al.*, 1994), we measured the effect of genistein on the efflux of Rh123. Figure 3b shows that genistein immediately accelerated the efflux of Rh123 from the resistant COR-L23/R cells. A similar efflux experiment was performed in MOR cells and genistein also accelerated the Rh123 efflux in the resistant cells of this line (Figure 4). The effects on Rh123 efflux were apparent within 5 min of administration of genistein in the resistant cell lines, whereas genistein reduced the retention of Rh123 in the parental cell lines significantly at time points beyond 90 min. Semi-logarithmic plotting of the retention data revealed that the efflux of Rh123 followed first-order kinetics in the resistant cells. Genistein enhanced the efflux of Rh123 3- to 5-fold in the resistant COR-L23/R and MOR/R cell lines, as well to some extent (<2-fold) in the parental MOR/P cells (Table I).

Next, we measured the concentration-dependent effect of genistein on Rh123 retention. Figure 5 shows a gradual decrease in Rh123 retention with increasing genistein concentrations in COR-L23/R cells with a maximal effect at 100–200 $\mu$M genistein. Only the highest genistein concentration had a significant effect in the COR-L23/P cells.

We then examined the effect of the other flavonoids on the retention of Rh123 and compared the effects with those of the resistance modifiers, verapamil, cyclosporin A and buthionine sulphoximine (BSO), as well as the cytotoxic agent vinblastine. Results for the COR-L23/R cells are shown in Figure 6. Treatment with BSO was given for 20 h before Rh123 retention was determined; the other modulators were added only during the efflux period. It can be seen that all modifiers, as well as the cytotoxic agent vinblastine, inhibited the efflux of Rh123 from the COR-L23/R cells. All the (iso)flavonoids tested decreased the retention of Rh123 in COR-L23/R cells, although genistin was only slightly active. Quercetin decreased the retention in the parental COR-L23/P cells to some extent, although less than in the resistant cells.

### *Effect of membrane potential on Rh123 transport*

Since the enhancement of the Rh123 efflux by the (iso)flavonoids is in contrast with the inhibition of the efflux of the cytotoxic agents, daunorubicin, doxorubicin and VP-16

**Figure 2** Modulation of daunorubicin (DNR) accumulation by (iso)flavonoids. Cells were incubated with 0.5 $\mu$M [$^3$H]daunorubicin for 60 min in the presence of 200 $\mu$M flavonoid or vehicle. Without modifer present, the daunorubicin accumulation in the resistant cell lines (▒) was decreased to 37%, 21% and 25% in the COR-L23/R (**a**), MOR/R (**b**) and H69/LX4 (**c**) cells, respectively, compared with the accumulation in their parental cell lines (□). Data are expressed as daunorubicin accumulation in the presence of modifier divided by daunorubicin accumulation with vehicle (0.5% DMSO) × 100%. Results are mean ± s.d. of at least three experiments.

**Figure 3** Effect of genistein on Rh123 transport in COR-L23 cells. (**a**) COR-L23/P (○,●) and COR-L23/R (△,▲) were exposed to 0.1 $\mu$g ml$^{-1}$ Rh123 in the presence of 200 $\mu$M genistein (●,▲) or vehicle (0.5% DMSO, ○,△). Each point is mean ± s.d. of three experiments. In each experiment, values were calculated relative to the fluorescence of the Rh123 accumulation in COR-L23/P cells at $t$ = 60 min, which was chosen as 100%. (**b**) For the efflux of Rh123, cells were incubated with Rh123 in the absence of genistein for 1 h, washed and resuspended in medium with (●,▲) or without (○,△) 200 $\mu$M genistein. Each point is mean ± s.d. of three experiments.

**Table I** Effect of genistein on $t_{1/2}$ of rhodamine 123 efflux

| | $t_{1/2}$ *(min)* Control | Genistein (200 μM) |
|---|---|---|
| COR-L23/P | >300 (3) | >200 (3) |
| COR-L23/R | 51±4 (3) | 11±3 (3)[a] |
| MOR/P | 185, 235 | 116, 119 |
| MOR/R | 25±4 (4) | 9±2 (3) |

[a]Data are significantly different from control, $P<0.02$, Student's *t*-test. Semi-logarithmic plotting of the retention data showed that the efflux of Rh123 from the resistant cells followed first-order kinetics (correlation coefficient $r^2=0.99$ during 2 h of Rh123 efflux or to 10% of the starting Rh123 content, whichever occurs first). Number of experiments in parentheses, except where only two experiments were carried out in which case individual values are shown.

(Versantvoort *et al.*, 1993), we considered the possibility that alterations in the accumulation of Rh123 rather than stimulation of the activity of the drug transporter causes the accelerated efflux of Rh123 by genistein. Because Rh123 depends for its accumulation on the mitochondrial membrane potential, we compared the effects of sodium azide, which is known to disrupt the mitochondrial membrane potential, with the effects of genistein. Sodium azide concentrations were chosen such that cellular ATP levels were not depleted to such a degree as to influence the transport of drugs (Versantvoort *et al.*, 1994). The effects of sodium azide on Rh123 efflux are shown in Figure 7. It can be seen that 25 mM sodium azide accelerated the efflux of Rh123 to a degree similar to the effect of genistein.

This effect of sodium azide might suggest that the acceleration of the Rh123 efflux by genistein is caused by alterations in the membrane potential. Therefore, we measured the membrane potential with the fluorescent probe

**Figure 4** Effect of genistein on Rh123 transport in MOR cells. MOR/P cells (○,●) and MOR/R cells (△,▲) were incubated with Rh123 in the absence of genistein for 1 h, washed and resuspended in medium with (●,▲) or without (○,△) 200 μM genistein. Each point is mean±s.d. of three experiments.

**Figure 5** Dose–response of genistein on Rh123 retention in COR-L23 cells. COR-L23/P (□) and COR-L23/R (▨) were loaded for 60 min with 0.1 μg ml$^{-1}$ Rh123 followed by efflux of Rh123 for 60 min. Results are mean±s.d. of at least three experiments.

**Figure 6** Effect of flavonoids and other modulators on Rh123 retention. Retention (60 min) of Rh123 was measured (**a**) in the presence of 10 μM verapamil (Vp), 4.2 μM cyclosporin A (CsA), 11 μM vinblastine (VBL), 200 μM genistein (GEN) and 25 μM buthionine sulphoximine (BSO, 20 h preincubation), or (**b**) in the presence of 200 μM genistein, genistin, quercetin and daidzein in COR-L23/P (□) and COR-L23/R cells (▨). Data are mean±s.d. of at least three experiments.

**Figure 7** Modulation of Rh123 efflux by sodium azide in COR-L23/R cells. Cells were incubated for 60 min with 0.1 $\mu$g ml$^{-1}$ Rh123 followed by an efflux of Rh123 in the absence (○) or presence of genistein (●) or 5 mM sodium azide (▲) or 25 mM sodium azide (■). Each point is mean ± s.d. of three experiments.

$DiOC_5$. The accumulation of $DiOC_5$ was rapid (steady state was reached in 15 min) and similar in the parental and the resistant COR-L23 cell lines (not shown). We then examined the effects of genistein on the accumulation of $DiOC_5$. As a comparison 100 $\mu$M DIDS, which is known to depolarise the membrane potential, was included in the experiments. DIDS, as well as genistein, decreased the accumulation of $DiOC_5$ to 60–72% of control in the COR-L23 cell lines. Depolarisation of the membrane potential by genistein was similar in parental and resistant cells, 66% and 60% respectively.

### Discussion

The plasma membrane protein P-gp is well known for its prominent role as a drug efflux pump in the MDR phenotype. Overexpression of MRP in tumour cell lines involves cross-resistance to similar cytotoxic drugs, such as daunorubicin, doxorubicin, vincristine, colchicine and etoposide, owing to an enhanced efflux of the drugs out of the cells (Zaman *et al.*, 1994; Grant *et al.*, 1994). Absolute discrimination between P-gp- and MRP-mediated resistance appears currently not to be achievable based on functional drug transport assays. However, the effects of various resistance modifiers vary considerably between the two types of MDR. Recently, we have shown that the isoflavonoid genistein and cellular glutathione depletion are potent inhibitors of MRP- but not P-gp-mediated daunorubicin transport (Versantvoort *et al.*, 1993, 1995). Moreover, Phang *et al.* (1993) showed that P-gp-mediated efflux was accelerated by flavonoids. Since glutathione depletion by buthionine sulphoximine takes several hours (Versantvoort *et al.*, 1995), genistein is potentially more useful in a functional assay to discriminate between P-gp- and MRP-mediated resistance.

In this study, the accumulation of daunorubicin was increased by genistein in the MRP-overexpressing MDR cell lines only (Figure 2), which is in accordance with our previous results (Versantvoort *et al.*, 1993). Of note was the reversal of the accumulation deficit of daunorubicin in the P-gp-overexpressing H69/LX4 cell line by genistin (Figure 2), since none of the (iso)flavonoids tested by Critchfield *et al.* (1994) was able to increase the accumulation of doxorubicin in the P-gp-expressing HCT-15 colon cells efficiently. The fact that genistin and genistein differ only by a glucose unit might have important implications, since many of the flavonoids found in fruits and vegetables are present as conjugates/glycosides (Hermann, 1976).

Furthermore, we were surprised by our finding that the efflux of Rh123 in the MRP-MDR cells was accelerated by genistein and the other (iso)flavonoids. This is in marked contrast to our previous results for daunorubicin, doxorubicin and VP-16 (Versantvoort *et al.*, 1993), indicating that the interaction between genistein and Rh123 is clearly different from that involving the cytotoxic drugs. We have shown in the $GLC_4$/ADR MRP-MDR cells that genistein is a competitive inhibitor of the daunorubicin efflux, indicating an interaction of genistein at the drug-binding site (Versantvoort *et al.*, 1994). The different effects of genistein might suggest that the drug-binding site at the transporter is different for daunorubicin and Rh123. Since other modifiers affect Rh123 transport in a similar way to the effects previously found for daunorubicin and vincristine transport (Barrand *et al.*, 1993), other mechanisms might evoke the acceleration of Rh123 transport in MRP-MDR cells.

An alternative mode of interaction between genistein and Rh123 was suggested by the observation that sodium azide, which lowers the mitochondrial membrane potential, was able to stimulate the efflux of Rh123 (Figure 7). The depolarisation of the membrane potential caused by genistein is then likely to affect the transport of Rh123. The different effects of genistein on the transport of Rh123 and daunorubicin in MRP-overexpressing MDR cells can be explained by the fact that Rh123, but not daunorubicin, is depending for its accumulation on the membrane potential. However, if depolarisation of the membrane potential rather than stimulation of MRP activity causes the alterations in Rh123 accumulation, it is then necessary to account for the different effects of genistein in parental and resistant COR-L23 cells, as depolarisation of the membrane potential by genistein was similar in the parental and resistant cells. The answer might be found in the different kinetics of Rh123 in the parental and resistant cells. Transport of Rh123 over the plasma membrane is determined by passive diffusion in the COR-L23/P cells and by a passive and active component in the COR-L23/R cells. Depolarisation of the membrane potential will affect passive as well as active transport of Rh123. Since the efflux of Rh123 is 6- to 10-fold faster from the resistant cells (active and passive transport) than from the parental cells (passive transport), the effects caused by depolarisation of the membrane potential will be apparent much faster in the resistant cells. As shown in Figures 3 and 4, the effects of genistein on Rh123 efflux are apparent in the resistant cells within 5 min of administration, whereas the effects on the parental cells were only significant at 120 min efflux or longer (data not shown).

It may be concluded from our results that the mechanism(s) by which flavonoids interact with drug transport is rather complex; not only was the daunorubicin transport mediated by P-gp and MRP affected differently by the flavonoids, but genistein and genistin had opposite effects on the daunorubicin transport mediated by P-gp, and the flavonoids had opposite effects on the transport of daunorubicin and Rh123 in MRP-overexpressing cells. These results show that the use of a more sensitive and/or cheaper probe (in this case Rh123) instead of the cytotoxic agent itself for selection of the most efficient resistance modifier must be regarded with caution, since the effects of modifiers on the probe may not predict for effects on cytotoxic agents.

### References

BARRAND MA, RHODES T, CENTER MS AND TWENTYMAN PR. (1993). Chemosensitisation and drug accumulation effects of cyclosporin A, PSC833 and verapamil in human MDR large cell lung cancer cells expressing a 190k membrane protein distinct from P-glycoprotein. *Eur. J. Cancer*, **29A,** 408-415.

BARRAND MA, HEPPEL-PARTON AC, WRIGHT KA, RABBITS PH AND TWENTYMAN PR. (1994). A 190k protein overexpressed in non-P-glycoprotein containing MDR cells and its relation to the MRP gene. *J. Natl Cancer Inst.*, **86,** 110-117.

CHAUDHARY PM AND RONINSON IB. (1991). Expression and activity of P-glycoprotein, a multidrug efflux pump, in human hematopoietic stem cells. *Cell*, **66,** 85-94.

COLE SPC, BHARDAWAJ G, GERLACH JH, MACKIE JE, GRANT CE, ALMQUIST KC, STEWART AJ, KURZ EU, DUNCAN AMV AND DEELEY RG. (1992). Overexpression of a novel transporter gene in a multidrug resistant human lung cancer cell line. *Science*, **258,** 1650-1654.

CRITCHFIELD JW, WELSH CJ, PHANG JM AND YEH GC. (1994). Modulation of adriamycin accumulation and efflux by flavonoids in HTC-15 colon cells. Activation of P-glycoprotein as a putative mechanism. *Biochem. Pharmacol.*, **48,** 1437-1445.

GOTTESMAN MM AND PASTAN I. (1993). Biochemistry of multidrug resistance mediated by the multidrug transporter. *Annu. Rev. Biochem.*, **62,** 385-427.

GRANT CE, VALDIMARSSON G, HIPFNER E, ALMQUIST KC, COLE SPC AND DEELEY RG. (1994). Overexpression of multidrug resistance-associated protein (MRP) increases resistance to natural product drugs. *Cancer Res.*, **54,** 337-361.

HERMANN K. (1976). Flavonols and flavones in food plants: a review. *J. Food. Technol.*, **11,** 433-448.

JEDLITSCHKY G, LEIER I, BUCHHOLZ U, CENTER MS AND KEPPLER D. (1994). ATP-dependent transport of glutathione *S*-conjugates by the multidrug resistance-associated protein. *Cancer Res.*, **54,** 4833-4836.

LUTZKY J, ASTOR MB, TAUB RN, BAKER MA, BHALLA K, GERVASONI Jr, JE, ROSADO M, STEWART V, KRISHNA S AND HINDENBURG AA. (1989). Role of glutathione and dependent enzymes in anthracycline-resistant HL60/AR cells. *Cancer Res.*, **49,** 4120-4125.

MÜLLER M, MEIJER C, ZAMAN GJR, BORST P, SCHEPER RJ, MULDER NH DE VRIES EGE AND JANSEN PLM. (1994). Overexpression of the gene encoding the multidrug resistance-associated protein results in increased ATP-dependent glutathione *S*-conjugate transport. *Proc. Natl Acad. Sci. USA*, **91,** 13033-13037.

PHANG JM, POORE CM, LOPACZYNSKA J AND YEH GC. (1993). Flavonol-stimulated efflux of 7,12-dimethylbenz(a)anthracene in multidrug resistant breast cancer cells. *Cancer Res.*, **53,** 5977-5981.

TWENTYMAN PR, FOX NE, WRIGHT KA AND BLEEHEN N. (1986). Derivation and preliminary characterisation of Adriamycin resistant lines of human lung cancer cells. *Br. J. Cancer*, **53,** 529-537.

TWENTYMAN PR, RHODES T AND RAYNER S. (1994). A comparison of rhodamine 123 accumulation and efflux in cells with P-glycoprotein-mediated and MRP-associated multidrug resistance phenotypes. *Eur. J. Cancer*, **30A,** 1360-1369.

SCHUURHUIS GJ, BROXTERMAN HJ, OSSEKOPPELE GJ, BAAK JPA, EEKMAN CA, KUIPER CM, FELLER N, VAN HEIJNINGEN THM, KLUMPER E, PIETERS R, LANKELMA J AND PINEDO HM. (1995). Functional multidrug resistance phenotype associated with combined overexpression of Pgp/MDR-1 and MRP together with 1-$\beta$-D-arabinofuranosylcytosine sensitivity may predict clinical response in acute myeloid leukemia. *Clin. Cancer Res.*, **1,** 81-93.

VERSANTVOORT CHM, SCHUURHUIS GJ, PINEDO HM, EEKMAN CA, KUIPER CM, LANKELMA J AND BROXTERMAN HJ. (1993). Genistein modulates the decreased drug accumulation in non-P-glycoprotein mediated multidrug resistant tumour cells. *Br. J. Cancer*, **68,** 939-946.

VERSANTVOORT CHM, BROXTERMAN HJ, LANKELMA J, FELLER N AND PINEDO HM. (1994). Competitive inhibition by genistein and ATP dependence of daunorubicin transport in intact *MRP* overexpressing human small cell lung cancer cells. *Biochem. Pharmacol.*, **48,** 1129-1136.

VERSANTVOORT CHM, BROXTERMAN HJ, BAGRIJ T, SCHEPER RJ AND TWENTYMAN PR. (1995). Regulation by glutathione of drug transport in multidrug-resistant human lung tumour cell lines overexpressing multidrug resistance-associated protein. *Br. J. Cancer*, **72,** 82-89.

ZAMAN GJR, FLENS MJ, VAN LEUSDEN MR, DE HAAS M, MÜLDER HS, LANKELMA J, PINEDO HM, SCHEPER RJ, BROXTERMAN HJ AND BORST P. (1994). The human multidrug resistance-associated protein MRP is a plasma membrane drug-efflux pump. *Proc. Natl Acad. Sci. USA*, **91,** 8822-8826.